# Construction and Immunogenicity of Modified Vaccinia Ankara (MVA) Viruses Expressing E1 and E2 Proteins of Bovine Viral Diarrhea Virus

**DOI:** 10.3390/vaccines14040337

**Published:** 2026-04-11

**Authors:** Yueyang Yu, Xiaohan Yan, Wenge Ma, Yuxin Liu, Zhiyi Liao, Xiaoyu Jiao, Pengpeng Wang, Chen Peng, Baifen Song, Wenxue Wu

**Affiliations:** National Key Laboratory of Veterinary Public Health and Safety, Key Laboratory of Animal Epidemiology of the Ministry of Agriculture and Rural Affairs, College of Veterinary Medicine, China Agricultural University, Beijing 100193, China; yuyueyang@cau.edu.cn (Y.Y.); yanxiaohan@cau.edu.cn (X.Y.); wenge_ma@163.com (W.M.); lyx19@cau.edu.cn (Y.L.); liaozhiyi0221@cau.edu.cn (Z.L.); bs20223050473@cau.edu.cn (X.J.); 18754881870@163.com (P.W.); pengchenea@cau.edu.cn (C.P.)

**Keywords:** modified vaccinia virus Ankara (MVA), bovine viral diarrhea virus (BVDV), glycoproteins E1 and E2, vaccine vector

## Abstract

**Background/Objectives:** Bovine viral diarrhea (BVD) is a major infectious disease of cattle caused by bovine viral diarrhea virus genotypes 1 and 2 (BVDV-1 and BVDV-2). Current inactivated and live attenuated vaccines provide incomplete cross-genotype protection and may exhibit limitations related to durability of immunity or safety. This study evaluated whether co-expression of the BVDV envelope glycoproteins E1 and E2 in a Modified Vaccinia Ankara (MVA) vector could support antigen expression and induce immune responses in a proof-of-concept model. **Methods:** Recombinant Modified Vaccinia Ankara (MVA) viruses expressing BVDV-1 E1E2 or BVDV-2 E1E2 were generated by homologous recombination. Recombinant viruses were purified and characterized for antigen expression, genetic stability, and growth properties in vitro. Immunogenicity was evaluated in a BALB/c mouse model by measuring E2-specific antibody responses, virus-neutralizing antibodies, and antigen-responsive cellular immune responses. **Results:** Both recombinant MVA constructs showed detectable E2 expression when E1 and E2 were co-expressed, and exhibited growth characteristics comparable to parental MVA with stable maintenance after serial passage. In contrast, recombinant MVA expressing E2 alone did not yield detectable E2 protein under the same experimental conditions. Immunization induced detectable humoral and cellular immune responses, including E2-specific IgG antibodies, virus-neutralizing antibodies, and increased frequencies of antigen-responsive CD8^+^ T cells with a tendency toward a Th1-biased profile. **Conclusions:** These findings indicate that co-expression of BVDV E1 and E2 in an MVA vector can support detectable antigen expression and induce measurable immune responses in a mouse proof-of-concept model. Further studies in cattle, including challenge experiments, will be required to determine the protective efficacy and practical applicability of this platform for BVDV vaccine development.

## 1. Introduction

Bovine viral diarrhea virus (BVDV) is one of the most important infectious agents affecting cattle, causing diarrhea, mucosal disease, reproductive disorders, and substantial economic losses worldwide [1]. In China, seroprevalence in unvaccinated cattle herds often exceeds 60–80% in some regions, highlighting the widespread circulation and significant impact of the virus [2,3]. BVDV belongs to the genus *Pestivirus* within the family *Flaviviridae*. According to the International Committee on Taxonomy of Viruses (ICTV), bovine viral diarrhea virus 1 and bovine viral diarrhea virus 2 are currently classified as two distinct species, *Pestivirus bovis* and *Pestivirus tauri*, respectively, and the two predominant genotypes circulating in cattle populations, BVDV-1 and BVDV-2, pose particular challenges for disease control [4,5]. Cross-protection between these two genotypes is often incomplete, which has motivated the development of vaccine strategies targeting both BVDV-1 and BVDV-2.

The viral genome is a positive-sense single-stranded RNA of approximately 12.3 kb containing a single open reading frame that encodes a large polyprotein precursor. This polyprotein is co- and post-translationally processed by viral and host proteases into structural proteins (C, Erns, E1, and E2) and several non-structural proteins. Among these structural proteins, the envelope glycoproteins E1 and E2 play essential roles in virion assembly, receptor interaction, and virus entry. During viral maturation, the E1–E2 region of the polyprotein is cleaved by host signal peptidase in the endoplasmic reticulum, and the two glycoproteins subsequently form heterodimers that are important for the proper folding and stability of E2 as well as for viral infectivity [4,6]. Functionally, E1 participates in viral replication and assembly and interacts closely with E2, whereas E2 serves as the principal antigenic determinant responsible for inducing neutralizing antibodies. Indeed, E2 has been extensively investigated as a vaccine antigen for BVDV over the past several decades, and a variety of recombinant E2-based vaccine approaches have been reported. However, several studies suggest that proper folding and antigenic presentation of E2 may depend on its interaction with E1, indicating that co-expression of E1 and E2 could improve antigen stability and immunogenicity compared with E2 expressed alone [7,8,9]. In addition, removal of the transmembrane region of E2 can generate a soluble form of the protein, which may facilitate antigen secretion and improve antigen accessibility without substantially affecting the major neutralizing epitopes located in the ectodomain [8,9,10].

Despite the availability of commercial vaccines, effective control of BVDV remains challenging. Current products consist mainly of inactivated BVDV-1 monovalent vaccines or combination vaccines formulated with other bovine respiratory pathogens such as infectious bovine rhinotracheitis virus (IBRV) and bovine parainfluenza virus type 3 (BPIV3) [11,12]. However, inactivated vaccines often provide only short-lived immunity, induce relatively weak cellular and mucosal immune responses, and show limited cross-protection against BVDV-2 despite some antigenic overlap. Live-attenuated vaccines, while generally more immunogenic, may raise safety concerns, including the potential risk of fetal infection, viral shedding, and reversion to virulence [13,14,15]. These limitations highlight the need for safer and more broadly protective vaccination strategies capable of inducing balanced humoral and cellular immune responses.

In recent years, recombinant live-vector vaccines expressing BVDV antigens have been explored using platforms such as Bacillus Calmette–Guérin (BCG), adenoviruses, and herpesviruses [16,17,18,19]. These studies have demonstrated that viral vectors expressing key BVDV antigens, particularly the E2 glycoprotein, can induce both humoral and cellular immune responses to varying degrees. Nevertheless, poxvirus-based BVDV vaccines remain relatively understudied. This is noteworthy because the modified vaccinia virus Ankara (MVA) strain is a highly attenuated vaccinia virus with a long record of safety in humans since its deployment as a smallpox vaccine in 1968 [20]. MVA combines host restriction, large foreign gene capacity, and strong induction of both humoral and cellular immune responses, and it has been widely applied as a vaccine vector against pathogens including SARS-CoV-2, chikungunya virus, hepatitis B and C viruses, and Zika virus [21,22,23,24,25]. Given that BVDV belongs to the same Flaviviridae family as hepatitis C and Zika viruses, MVA represents a promising yet largely unexplored platform for BVDV vaccine development.

In this study, we generated modified vaccinia virus Ankara (MVA) constructs co-expressing the BVDV E1 glycoprotein and a truncated soluble form of E2 derived from both BVDV-1 and BVDV-2. Two recombinant viruses were constructed, rMVA-BVDV1-E1E2 and rMVA-BVDV2-E1E2. For simplicity, these constructs are collectively referred to as rMVA-E1E2 vaccines unless otherwise specified. Using homologous recombination at the MVA069R–MVA070L locus, recombinant viruses were constructed and characterized for genetic stability and growth properties. The immunogenicity of these recombinant viruses was evaluated in a BALB/c mouse model by assessing neutralizing antibody responses and antigen-specific cellular immunity. These results provide a preliminary evaluation of MVA-vectored E1E2 vaccine candidates and suggest that this platform warrants further investigation for BVDV vaccine development.

## 2. Materials and Methods

### 2.1. Cells

BHK-21 and DF-1 cells were obtained from the China Veterinary Culture Collection Center (CVCC, Beijing, China) and maintained in Dulbecco’s Modified Eagle Medium (DMEM; HyClone, Cytiva, Logan, UT, USA) supplemented with 10% heat-inactivated fetal bovine serum (FBS; VivaCell Biosciences, Shanghai, China) and 1% penicillin–streptomycin (Solarbio, Beijing, China). Cells were incubated at 37 °C in a humidified atmosphere containing 5% CO_2_.

### 2.2. Viruses

The Modified Vaccinia Virus Ankara (MVA) strain was kindly provided by Dr. Bernard Moss (National Institutes of Health, USA). BVDV-1 and BVDV-2 strains were obtained from the Rapid Diagnostic Techniques Laboratory for Animal Diseases at China Agricultural University.

### 2.3. Antibodies

A BVDV-1-specific monoclonal antibody (157, BVDV-1 E2 gp53, P200511-001) and a BVDV-2-specific monoclonal antibody (BA-2, P200511-001) were purchased from VMRD^®^ (Pullman, WA, USA). The BVDV-1-specific monoclonal antibody (3G3) was generated in our laboratory. Vaccinia virus (VACV)-positive serum was kindly provided by Dr. Bernard Moss (National Institutes of Health, USA).

### 2.4. Construction of Recombinant Transfer Vector

DNA sequences encoding the E1E2 proteins of BVDV-1 and BVDV-2 were synthesized, with C-terminal fusion of Myc and 3 × Flag tags, respectively. The E2 coding sequence was codon-optimized and truncated to remove the transmembrane and intracellular domains to facilitate expression.

The transfer cassette was designed to target the MVA069R–MVA070L locus. Homologous arms corresponding to MVA069R (nt 63,440–63,809) and MVA070L (nt 63,841–64,269) flanked a gene cassette containing, in sequence, the pmH5 promoter, the E1E2 gene, a LoxP site, the p11 promoter, the mCherry reporter gene, and a second LoxP site. Recombinant transfer plasmids pUC19-rMVA-BVDV1-E1E2 and pUC19-rMVA-BVDV2-E1E2 were synthesized by Sangon Biotech Co., Ltd. (Shanghai, China).

### 2.5. Construction of Recombinant Viruses

BHK-21 cells seeded in 6-well plates were grown to approximately 80–90% confluence and infected with parental MVA at a multiplicity of infection (MOI) of 3 in maintenance medium (DMEM supplemented with 2.5% FBS). After 2 h of adsorption, cells were washed with phosphate-buffered saline (PBS).

Recombinant transfer plasmids were transfected using Effectene Transfection Reagent (Qiagen, Hilden, Germany) according to the manufacturer’s instructions. Briefly, plasmid DNA was mixed with Enhancer and Buffer EC to form DNA–lipid complexes, which were added dropwise to infected cells. After incubation, fresh maintenance medium was added, and cells were cultured for 24–48 h. Cultures exhibiting mCherry fluorescence were harvested as Passage 0 (P0).

Virus lysates were prepared by three freeze–thaw cycles and sonication, followed by centrifugation at 2500 rpm for 5 min. Serial dilutions were used to infect fresh BHK-21 cells, which were overlaid with carboxymethyl cellulose-containing medium for plaque purification. Individual mCherry-positive plaques were isolated and subjected to successive rounds of purification. After 5–7 passages, viral DNA was extracted and analyzed by PCR using primers flanking the MVA069R and MVA070L regions: Forward: 5′-tttggatattctatggcgtacaaaggaata-3′; Reverse: 5′-cattttttgctagtggtaattccatagatg-3′. PCR products were confirmed by Sanger sequencing.

### 2.6. Characterization of rMVA-BVDV1-E1E2 and rMVA-BVDV2-E1E2

The growth properties of rMVA-BVDV1-E1E2 and rMVA-BVDV2-E1E2 were evaluated using a one-step growth curve assay. DF-1 and BHK-21 cells were infected with rMVA-E1E2 or parental MVA at a multiplicity of infection (MOI) of 0.01. Cell-associated virus was harvested at 0, 24, 48, and 72 h post-infection, subjected to three freeze–thaw cycles followed by sonication, and titrated on DF-1 cells.

For ultrastructural analysis, BHK-21 cells were infected with either parental MVA or rMVA-E1E2 at an MOI of 10 PFU/cell. At 24 h post-infection, cells were fixed with 2.5% glutaraldehyde in 0.1 M acetate buffer, either at room temperature for 2–3 h or at 4 °C overnight. Fixed samples were embedded in Eponate 12 resin, sectioned into 80-nm ultrathin slices, and stained with 5% uranyl acetate and 2% lead citrate. Virion morphology was examined using a JEOL 1200EX transmission electron microscope (JEOL, Tokyo, Japan).

To assess genetic stability, rMVA-BVDV1-E1E2 and rMVA-BVDV2-E1E2 were serially passaged for 20 consecutive generations in DF-1 cells. Viral DNA was extracted from each passage and subjected to PCR amplification using the primers described in Section 2.5. The resulting PCR products were sequenced by Sangon Biotech to verify the integrity of the E1E2 insert.

### 2.7. Western Blotting Analysis

BHK-21 cells were thawed from liquid nitrogen and cultured until stable growth was established. At 80% confluency in 6-well plates, cells were infected with rMVA-E1E2 at an MOI of 3 for 24 h. Control groups included parental MVA-infected cells (negative control) and uninfected cells (mock control).

Following infection, cells were washed with PBS and lysed on ice with 100 µL NP-40 or RIPA buffer containing 1 mM PMSF. Lysates were centrifuged at 12,000 rpm for 10 min at 4 °C, and supernatants were mixed with 6× protein loading buffer and boiled at 100 °C for 10 min.

Proteins were separated by sodium dodecyl sulfate–polyacrylamide gel electrophoresis (SDS-PAGE) using either 4–20% gradient FastPAGE precast gels (Tsingke Biotechnology, Beijing, China) or handcast gels consisting of a 10% separating gel and a 5% stacking gel (Beyotime, Shanghai, China). The membrane was blocked with 5% skim milk for 2 h at room temperature, then incubated overnight at 4 °C with mouse anti-BVDV-1 (3G3) or anti-BVDV-2(BA-2) monoclonal antibodies (1:2000). After washing, membranes were incubated with HRP-conjugated goat anti-mouse IgG (1:5000; Beyotime, Shanghai, China) for 1 h at room temperature. Protein bands were visualized using ECL substrate (Tanon, Shanghai, China) and imaged with a gel documentation system.

### 2.8. Detection of E2 Protein by Immunofluorescence Assay

BHK-21 cells were cultured in 6-well plates and grown to 80% confluency before infection with rMVA-BVDV1-E1E2 or rMVA-BVDV2-E1E2 at an MOI of 3 in maintenance medium for 24 h. Control wells were inoculated with parental MVA (negative control) or left uninfected (mock control).

Following incubation, cells were washed with PBS and subjected to the following steps: fixation with 4% paraformaldehyde (30 min, room temperature), permeabilization with 0.2% Triton X-100 (20 min, room temperature), and blocking with 1% BSA (1 h, 37 °C). The cells were then incubated sequentially with mouse anti-BVDV-1(157) or anti-BVDV-2 (BA-2) monoclonal antibodies (1:500, 1.5 h, 37 °C) and FITC-conjugated goat anti-mouse IgG (1:1000, 1 h, 37 °C, Beyotime, Shanghai, China). Nuclei were counterstained with DAPI (1:100 in blocking buffer, 10 min, room temperature, Beyotime, Shanghai, China). After final washing, fluorescence was examined using a fluorescence microscope (Olympus, Tokyo, Japan).

### 2.9. Immunization of Mice

The animal ethics committee of China Agricultural University has approved this immunogenicity study. All experimental procedures involving animals were conducted in accordance with international guidelines and relevant Chinese laws and regulations.

Female BALB/c mice aged 6–7 weeks were divided into six groups (*n* = 10 per group) and administered either a high dose [2 × 10^8^ PFU/100 µL,50 µL] or a low dose [2 × 10^7^ PFU/100 µL,50 µL] via the intranasal or intramuscular route. A commercially available inactivated BVDV vaccine (BVDV type 1, NM01 strain; Tianfujing^®^, Tiankang Biological Co., Ltd., Urumqi, China) was used as the inactivated vaccine control. An MVA-negative control group was also included. All mice received a booster immunization 14 days after the primary immunization.

### 2.10. Neutralizing Antibody Assay

Sera collected from immunized and control mice were heat-inactivated at 56 °C for 30 min. Serial two-fold dilutions of each serum sample were prepared in Dulbecco’s modified Eagle’s medium (DMEM) supplemented with 2% horse serum using a 96-well U-bottom plate (0.1 mL per well) in duplicate. Virus suspensions of BVDV-1 and BVDV-2 were diluted to 200 TCID_50_/0.1 mL in the same medium, and equal volumes of virus suspension were mixed with each diluted serum sample.

For cross-neutralization analysis, sera from mice immunized with rMVA-BVDV1-E1E2 were also tested against BVDV-2, whereas sera from mice immunized with rMVA-BVDV2-E1E2 were tested against BVDV-1.

The serum–virus mixtures were incubated at 37 °C with 5% CO_2_ for 1 h to allow virus–serum neutralization. MDBK cells seeded in 96-well plates 16–24 h earlier were washed with phosphate-buffered saline (PBS), and 0.1 mL of the serum–virus mixture was added to each well of the cell monolayer (four replicates per sample). The plates were incubated at 37 °C with 5% CO_2_ for 2–3 days and monitored for cytopathic effects (CPE).

The neutralization titer was defined as the highest serum dilution that completely prevented CPE in MDBK cells.

### 2.11. Detection of E2-Binding Antibodies by ELISA

To characterize the humoral immunity elicited by rMVA-BVDV1-E1E2 and rMVA-BVDV2-E1E2, serum levels of BVDV E2-specific IgG and its subtypes were determined by indirect ELISA. Purified BVDV E2 protein (0.1 μg/mL), produced in our laboratory using a baculovirus expression vector system and purified by Ni-affinity chromatography as previously described [26], was coated onto ELISA plates (100 μL per well) and incubated overnight at 4 °C. After removing the liquid, wells were washed three times with 300 μL PBST (PBS with 0.05% Tween-20) under shaking for 3 min each, then tapped dry. Blocking was carried out with 5% skim milk (200 μL/well) for 2 h at 37 °C. Serum samples were serially diluted two-fold from an initial 1:2 dilution up to 1:2^n^. After washing with PBST, 100 μL of each dilution was added to the plate and incubated at 37 °C for 1.5 h. Following another wash, wells were incubated with HRP-conjugated goat anti-mouse IgG (1:5000 in blocking buffer, 100 μL/well, Beyotime, Shanghai, China), IgG1 or IgG2a (abclonal, Wuhan, China) for 1 h at 37 °C. After a final wash, 100 μL TMB substrate (Solarbio, Beijing, China) was added and developed for 10 min at 37 °C. The reaction was terminated with 50 μL stop solution (2 M H_2_SO_4_), and the absorbance was read at 450 nm (Tecan, Männedorf, Switzerland). The antibody titer was defined as the highest serum dilution satisfying all of the following: sample OD_450 nm_ > 1, negative control OD_450 nm_ < 0.1, and P/N ratio ≥ 2.1.

### 2.12. Antigen-Specific T Cell Stimulation and Analysis by Flow Cytometry and ELISpot

To assess cellular immune responses induced by rMVA-BVDV1-E1E2 and rMVA-BVDV2-E1E2, antigen-specific T cell activation in splenic lymphocytes was evaluated by flow cytometry and IFN-γ ELISpot assay. Spleens were harvested from immunized mice and mechanically dissociated through a 70-μm cell strainer to obtain single-cell suspensions. Following erythrocyte lysis with cold ammonium–chloride–potassium buffer, splenocytes were washed, resuspended in RPMI-1640 medium (HyClone, Cytiva, Logan, UT, USA), and counted. Cells were adjusted to a concentration of 1.0 × 10^7^ cells/mL and stimulated with purified BVDV E2 protein (final concentration, 10 μg/mL) in 96-well plates for 6 h at 37 °C. The purified BVDV E2 protein used for stimulation was prepared as described in Section 2.11.

For flow cytometric analysis, cells were incubated with anti-CD16/32 antibody (Invitrogen, Carlsbad, CA, USA) to block Fc receptors and subsequently stained with fluorochrome-conjugated antibodies against surface markers, including FITC-anti-mouse CD3, PE-anti-mouse CD4, and PerCP/Cy5.5-anti-mouse CD8a (all from BioLegend, San Diego, CA, USA), for 1 h at 4 °C in the dark. After washing with staining buffer (eBioscience, San Diego, CA, USA), cells were fixed and permeabilized using the Cytofix/Cytoperm kit according to the manufacturer’s instructions and analyzed on a BD Verse flow cytometer (BD Biosciences, San Jose, CA, USA).

In parallel, E2-stimulated splenocytes were subjected to an IFN-γ ELISpot (DAKEWE, Shenzhen, China) assay to quantify antigen-specific T cell responses. Cells were seeded into wells of a mouse IFN-γ pre-coated ELISpot plate at a density of 1.0 × 10^6^ cells/mL (100 μL per well). The assay was performed following the manufacturer’s protocol, and spot-forming cells were enumerated as an indicator of IFN-γ–secreting T cell frequency.

### 2.13. Statistical Analysis

All data analyses were carried out using GraphPad Prism 6 software. Results are expressed as mean ± standard deviation (SD). Statistical analysis was performed by two-way analysis of variance (ANOVA) with Tukey’s multiple-comparison test in GraphPad Prism.

## 3. Results

### 3.1. Construction and Purification of rMVA-BVDV1-E1E2 and rMVA-BVDV2-E1E2

**Figure 1:**
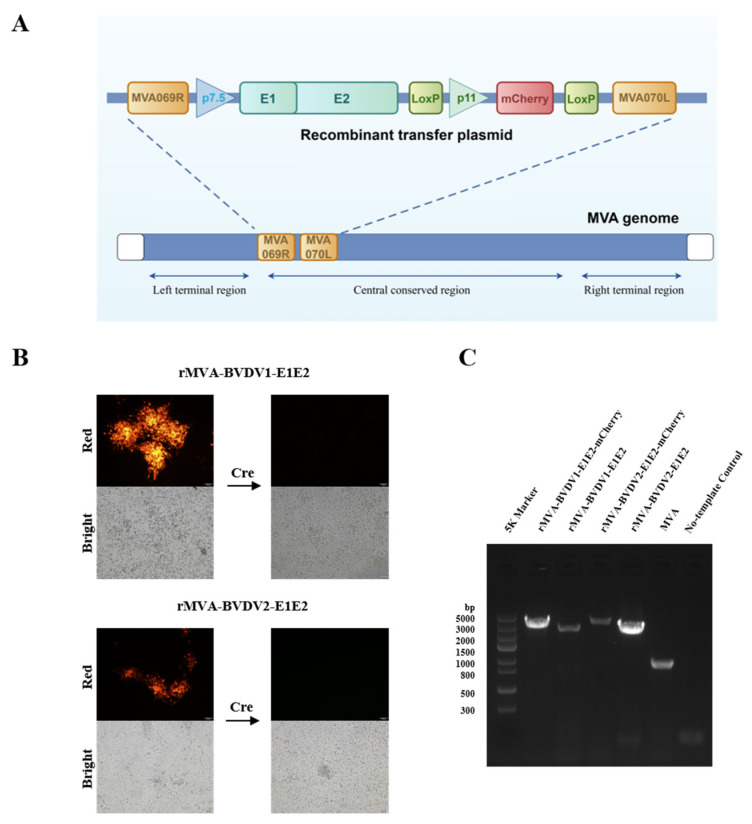
Construction of rMVA-E1E2: (**A**) Schematic of the rMVA-E1E2 engineering strategy. A gene cassette containing the P7.5 promoter-driven *E1E2* gene and the P11 promoter-driven *mCherry* reporter was inserted between the *MVA067R* and *MVA070F* loci. The *mCherry* gene, flanked by CRE-recognition sites (loxP), was subsequently excised via Cre-loxP recombination. (**B**) Fluorescence microscopy images of BHK-21 cells infected with rMVA-E1E2 before (**left**) and after (**right**) mCherry knockout. (**C**) PCR confirmation of the *E1E2* gene insertion and the successful removal of the *mCherry* cassette.

Recombinant MVA expressing BVDV E1E2 was generated by infecting BHK-21 cells with parental MVA followed by transfection with the recombinant transfer plasmid pUC19-rMVA-E1E2, as schematically illustrated in Figure 1A. Homologous recombination between the viral genome and the transfer plasmid occurred at the MVA069R–MVA070L locus during viral replication. The mCherry reporter gene enabled rapid identification and selection of recombinant viral clones by fluorescence microscopy, which were subsequently subjected to serial plaque purification.

After 4–6 rounds of purification, viral DNA was extracted and screened by PCR using primers flanking the recombination locus. Following transient expression of Cre recombinase and additional purification rounds, recombinant viruses lacking the mCherry marker were obtained (Figure 1B). PCR analysis revealed a fragment of approximately 900 bp for parental MVA, whereas larger fragments of approximately 3339 bp and 3441 bp were detected for rMVA-BVDV1-E1E2 and rMVA-BVDV2-E1E2, respectively (Figure 1C). The absence of the parental MVA-specific band in recombinant virus preparations indicated successful removal of residual parental virus. Sanger sequencing further confirmed correct insertion of the E1E2 expression cassette, demonstrating successful rescue of rMVA-BVDV1-E1E2 and rMVA-BVDV2-E1E2.

### 3.2. Expression of BVDV E2 Protein in BHK-21 Cells Infected with rMVA-BVDV1-E1E2 and rMVA-BVDV2-E1E2

**Figure 2:**
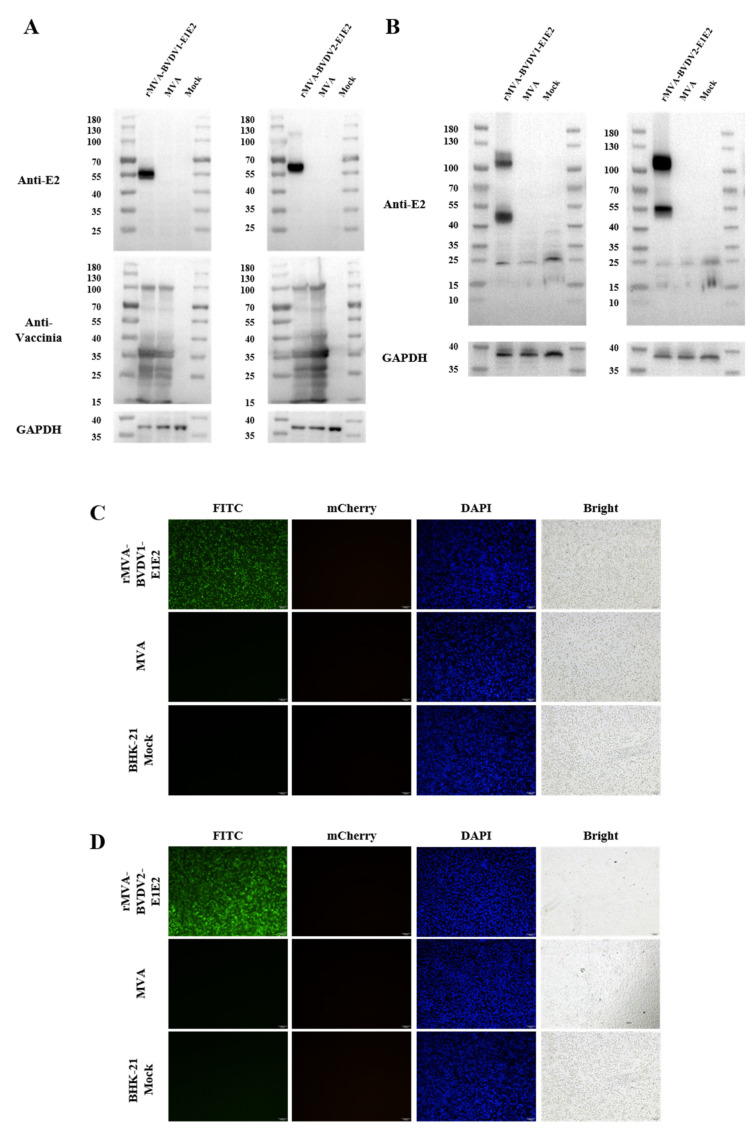
Expression of the E2 protein in recombinant MVA. (**A**) Western blot analysis. BHK-21 cells were mock-infected or infected with purified parental MVA or rMVA-E1E2 at 3 PFU/cell for 24 h. Cell lysates were subjected to SDS-PAGE and immunoblotted with antibodies specific for BVDV E2 and GAPDH, or with vaccinia virus (VACV)-positive serum. (**B**) BHK-21 cells were mock-infected or infected with purified parental MVA or rMVA-E1E2 at 3 PFU/cell for 24 h, and lysates were analyzed under non-reducing conditions by native-PAGE and Western blotting analysis using BVDV E2 and GAPDH antibodies. E2 monomer and E2 dimer are shown in (**B**). (**C**,**D**) Immunofluorescence analysis. At 24 h post-infection under the same conditions as in (**A**), cells were fixed, permeabilized, and blocked. Intracellular E2 was detected using specific primary antibodies followed by FITC-conjugated secondary antibodies (green). Nuclei were counterstained with Hoechst (blue).

Expression of the recombinant E2 protein was examined in BHK-21 cells infected with rMVA-E1E2. Western blot analysis detected specific protein bands of approximately 55 kDa and 56 kDa corresponding to Myc-tagged BVDV-1 E2 and 3×Flag-tagged BVDV-2 E2, respectively (Figure 2A). These bands were specifically recognized by BVDV E2-specific monoclonal antibodies, confirming efficient expression of the recombinant proteins. In contrast, recombinant MVA constructs expressing E2 alone did not yield detectable E2 protein when analyzed by Western blotting using BVDV E2-specific monoclonal antibodies; moreover, the construct expressing BVDV-1 E2 could not be detected even with BVDV-1 polyclonal antibodies (Supplementary Figure S1).

We next assessed the oligomeric state of the E2 glycoprotein using non-reducing native-PAGE. As shown in Figure 2B, two bands were observed on the gel: a predominant band corresponding to the E2 dimer at approximately 100 kDa, and a fainter band corresponding to the E2 monomer at approximately 55 kDa (see Figure S2 for the original uncropped blots). The detection of dimeric E2 under native conditions is consistent with the known oligomeric organization of pestiviral envelope glycoproteins.

Consistent with these findings, indirect immunofluorescence assays performed in infected BHK-21 cells demonstrated distinct cytoplasmic fluorescence signals when probed with BVDV-1- or BVDV-2-specific monoclonal antibodies, whereas no specific signal was observed in control cells (Figure 2C,D; see Figures S3 and S4 for the original images). Together, these results verify that rMVA-BVDV1-E1E2 and rMVA-BVDV2-E1E2 direct robust and antigen-specific expression of BVDV E2 in BHK-21 cells, whereas no specific signal was detected in control cells infected with parental MVA or in mock-infected cells.

### 3.3. Characterization of rMVA-BVDV1-E1E2 and rMVA-BVDV2-E1E2 Recombinant Viruses

#### 3.3.1. Growth Kinetics of rMVA-BVDV1-E1E2 and rMVA-BVDV2-E1E2 Viruses

**Figure 3:**
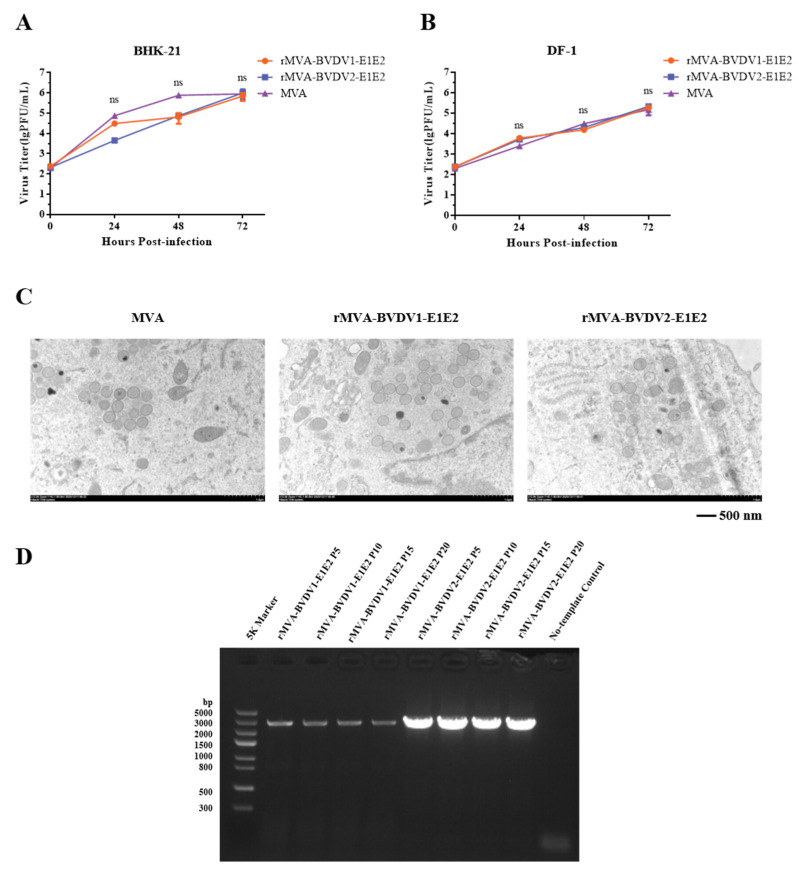
Biological characterization of rMVA-BVDV1-E1E2 and rMVA-BVDV2-E1E2. (**A**,**B**) Growth kinetics. BHK-21 (**A**) or DF-1 (**B**) cells were infected with parental MVA or rMVA-E1E2 at an MOI of 0.01. Cell-associated viruses were harvested at the indicated time points (24, 48, and 72 h post-infection), and titers were determined by plaque assay on DF-1 cells. (**C**) Virion morphology. BHK-21 cells infected with parental MVA or rMVA-E1E2 (MOI = 10) for 24 h were analyzed by transmission electron microscopy (TEM). (**D**) Genetic stability. The integrity of the *E1E2* gene insert in rMVA-E1E2 after 20 serial passages was assessed by PCR.

To assess whether insertion of the E1E2 expression cassette affected viral replication, one-step growth curves were determined in DF-1 and BHK-21 cells. Cells were infected with parental MVA or rMVA-E1E2 at 0.01 MOI, and viral titers were measured at 24, 48 and 72 h after infection. As shown in Figure 3A,B, both parental and recombinant viruses exhibited comparable growth kinetics in the two cell lines, with viral titers increasing over time and reaching peak levels at 72 h post-infection. No statistically significant differences were observed between rMVA-E1E2 and parental MVA, indicating that E1E2 gene insertion does not impair viral replication in permissive cells.

#### 3.3.2. Observation of rMVA-BVDV1-E1E2 and rMVA-BVDV2-E1E2 Virion Morphology

Transmission electron microscopy was used to examine the morphology of rMVA-BVDV1-E1E2 and rMVA-BVDV2-E1E2 in infected BHK-21 cells. As shown in Figure 3C, cells infected with either rMVA-E1E2 or parental MVA contained spherical immature virions with diameters of approximately 300 nm. No obvious morphological differences were observed between recombinant and parental viruses. These observations are consistent with the known replication characteristics of MVA in permissive cells and indicate that recombinant construction did not alter virion morphology under the conditions tested.

#### 3.3.3. Genetic Stability of rMVA-BVDV1-E1E2 and rMVA-BVDV2-E1E2

The genetic stability of rMVA-BVDV1-E1E2 and rMVA-BVDV2-E1E2 was evaluated by serial passaging in DF-1 cells for up to 20 passages. PCR amplification of viral DNA confirmed the persistent presence of the E1E2 gene after serial passaging (Figure 3D). Sequencing of the E1E2 region from the 10th and 20th passages revealed no nucleotide substitutions compared with the original construct. These results demonstrate that rMVA-E1E2 maintains genetic stability during extended propagation in vitro.

### 3.4. rMVA-BVDV1-E1E2 and rMVA-BVDV2-E1E2 Induce Humoral and Cellular Immune Responses

**Figure 4:**
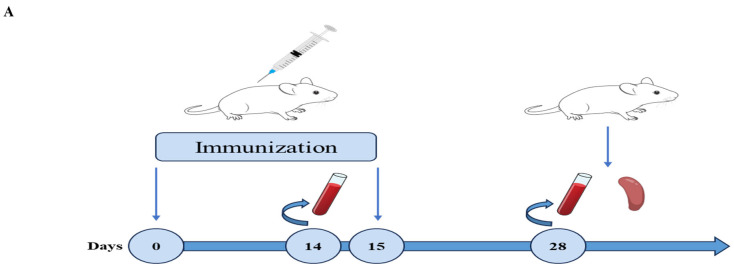

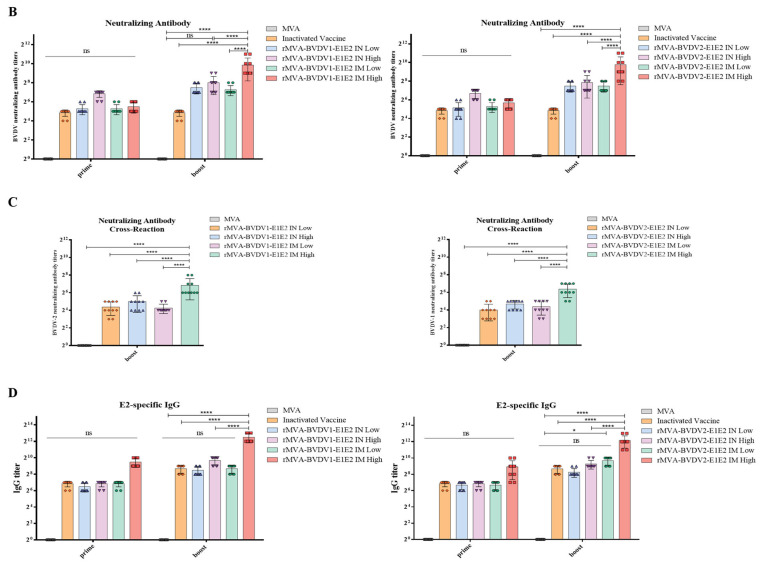

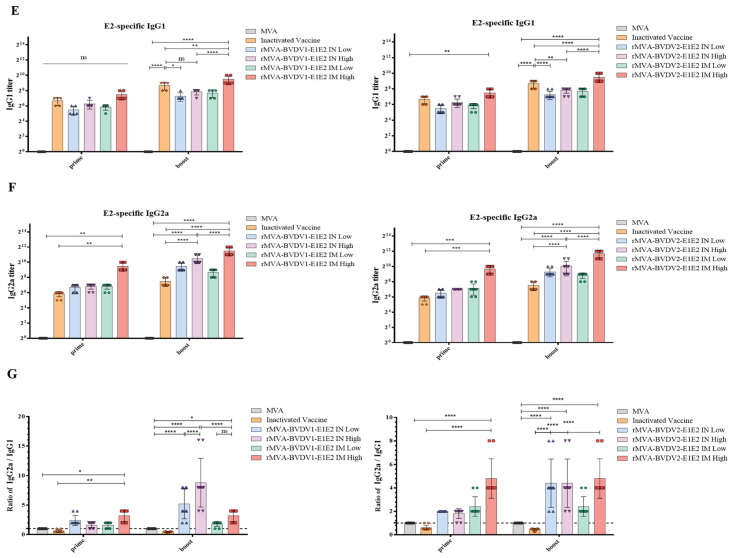
Humoral immune responses induced by MVA-vectored BVDV vaccines. (**A**) Immunization schedule. Schematic representation of the mouse immunization and sample collection timeline. (**B**) Homologous virus-neutralizing antibodies. Serum-neutralizing antibody titers against homologous BVDV strains were measured using sera collected 2 weeks post-primary and post-booster immunization. (**C**) Heterologous virus-neutralizing antibodies. Serum-neutralizing antibody titers against heterologous BVDV strains were determined by virus neutralization assay using sera collected 2 weeks after booster immunization. (**D**) E2-specific IgG antibodies. Levels of E2-binding IgG in serum collected before vaccination and at 2 weeks post-primary and post-booster immunization were determined by ELISA. (**E**,**F**) IgG subclass analysis. Antigen-specific IgG2a (**E**) and IgG1 (**F**) levels were quantified by ELISA. (**G**) IgG2a/IgG1 ratio. The ratio of IgG2a to IgG1 was calculated to evaluate the Th1/Th2 bias of the immune response. Data are presented as mean ± SD (n = 10). Statistical significance was analyzed using two-way ANOVA followed by Tukey’s multiple comparison test. ns, no significant, * *p* < 0.05, ** *p* < 0.01, *** *p* < 0.001, **** *p* < 0.0001.

To evaluate the immunogenicity of rMVA-E1E2, 6–7-week-old BALB/c mice were immunized via either the intranasal (IN) or intramuscular (IM) route using two dose levels (10^8^ PFU or 10^7^ PFU). A homologous booster immunization was administered 14 days after the primary dose. Serum samples were collected 14 days after primary and booster immunization for humoral immune analysis, and splenocytes were harvested after boosting for cellular immune assessment (Figure 4A).

Neutralizing antibody (NAb) responses against BVDV were evaluated using a virus neutralization assay recommended by the World Organization for Animal Health (OIE). As shown in Figure 4B, mice immunized with rMVA-BVDV1-E1E2 and rMVA-BVDV2-E1E2 developed detectable neutralizing antibodies against homologous BVDV strains. In both immunization routes, the high-dose groups generally produced higher neutralizing antibody titers than the corresponding low-dose groups. Intramuscular (IM) immunization tended to induce higher neutralizing titers than intranasal (IN) administration. Compared with the MVA control group, rMVA-E1E2–immunized mice showed significantly increased NAb titers, while the titers were broadly comparable to those observed in the inactivated vaccine group.

To further assess cross-neutralizing activity, sera collected after booster immunization were tested against heterologous BVDV strains. As shown in Figure 4C, sera from rMVA-E1E2–immunized mice were able to neutralize heterologous BVDV (rMVA-BVDV1-E1E2 vaccine against BVDV-2 and rMVA-BVDV2-E1E2 vaccine against BVDV-1), although the titers were generally lower than those observed against homologous viruses (approximately 4–32-fold lower in this study). Nevertheless, cross-neutralizing antibodies were detectable in both IM and IN immunization groups. Similar to the homologous neutralization results, higher titers were generally observed in the high-dose groups and in mice immunized via the IM route.

Consistent with the neutralization data, E2-specific IgG antibody levels measured by indirect ELISA increased after vaccination (Figure 4D). Among rMVA-E1E2–immunized mice, the highest IgG titers were observed in the high-dose IM group. Overall, IgG levels in vaccinated mice were higher than those in the MVA control group and were comparable to those observed in the inactivated vaccine group.

To further characterize the antibody response, IgG subclass distributions were analyzed by ELISA. Immunization with rMVA-E1E2 resulted in higher levels of IgG2a than IgG1 in most experimental groups, leading to IgG2a/IgG1 ratios greater than 1 (Figure 4E–G). This pattern suggests a tendency toward a Th1-biased immune response, which is commonly observed in viral vector vaccines.

**Figure 5:**
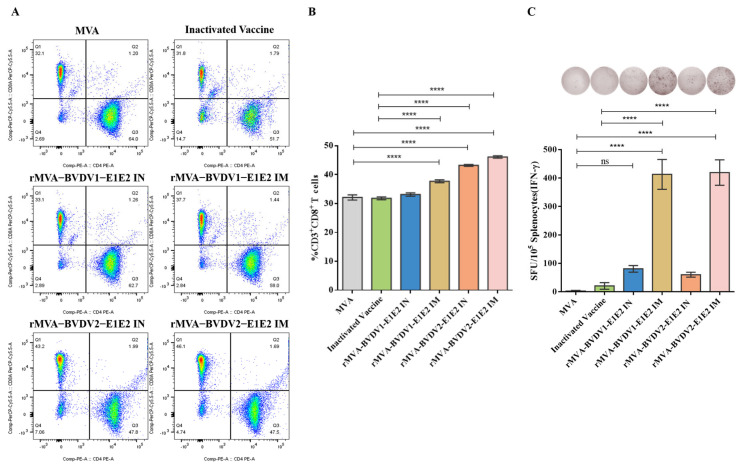
Cellular immune responses induced by rMVA-BVDV1-E1E2 and rMVA-BVDV2-E1E2 vaccines in mice. (**A**) Flow cytometry analysis. Representative gating strategy used to identify T-cell populations in mouse splenocytes after in vitro stimulation with BVDV E2 protein. (**B**) Frequency of CD8^+^ T cells. Percentage of CD3^+^CD8^+^ T cells among total CD3^+^ lymphocytes determined by flow cytometry. (**C**) IFN-γ ELISpot assay. Splenocytes isolated from immunized mice were stimulated with BVDV E2 protein, and IFN-γ-secreting cells were enumerated by ELISpot. Data are presented as spot-forming units (SFU) per 10^5^ splenocytes. Data are shown as mean ± SD. Statistical significance was determined using two-way ANOVA followed by Tukey’s multiple comparison test. * *p* < 0.05, ** *p* < 0.01, *** *p* < 0.001, **** *p* < 0.0001.

Cellular immune responses were subsequently evaluated by flow cytometry following in vitro stimulation of splenocytes with BVDV E2 protein. The proportion of CD8^+^ T cells among CD3^+^ lymphocytes was increased in rMVA-E1E2–immunized mice compared with the MVA control group (Figure 5A,B), suggesting an increased frequency of CD8^+^ T cells following immunization.

In addition, IFN-γ ELISpot assays detected higher frequencies of IFN-γ–secreting T cells in splenocytes from rMVA-E1E2–immunized mice following E2 stimulation compared with those from control groups (Figure 5C). No IFN-γ–secreting cells were detected in the MVA control group after antigen stimulation. Together, these results indicate that rMVA-E1E2 vaccination induced measurable humoral and cellular immune responses in mice.

## 4. Discussion

Bovine viral diarrhea virus remains a major threat to cattle health worldwide, causing substantial economic losses through reproductive failure, immunosuppression, and increased susceptibility to secondary infections [27,28]. Although vaccination has been widely implemented, effective disease control is hindered by the genetic diversity of BVDV and the intrinsic limitations of currently available vaccines [29,30,31]. In this study, we developed a recombinant Modified Vaccinia Virus Ankara platform co-expressing the BVDV E1 and E2 glycoproteins and systematically evaluated its biological characteristics and immunogenicity in a mouse proof-of-concept model, with the aim of providing an initial assessment of this platform as a potential vaccine strategy rather than establishing protective efficacy.

E2 is widely recognized as the principal target of virus-neutralizing antibodies and has therefore been the primary focus of most BVDV subunit and vector-based vaccine strategies [32,33,34,35,36]. However, increasing evidence indicates that the expression, stability, and antigenicity of E2 are strongly dependent on its structural context. In our preliminary experiments, recombinant MVA constructs expressing BVDV E2 alone did not yield detectable E2 protein when probed with BVDV E2–specific monoclonal antibodies by Western blotting. Moreover, the recombinant MVA expressing BVDV-1 E2 could not be detected even when probed with BVDV-1 polyclonal antibodies. In contrast, when E1 and E2 were co-expressed, the E2 protein became detectable in infected cells. This observation suggests that the presence of E1 may facilitate the detectable expression or stabilization of E2 in the recombinant system, which is consistent with previous reports that E1 contributes to correct folding and heterodimer formation of pestiviral envelope glycoproteins [37,38,39,40,41,42,43]. Importantly, the major neutralizing epitopes of BVDV E2 are located within the ectodomain, suggesting that removal of the transmembrane region is unlikely to substantially alter the key antigenic determinants recognized by neutralizing antibodies. Accordingly, these findings should be interpreted as evidence supporting the structural interdependence of E1 and E2 rather than demonstrating improved immunogenicity of the vaccine construct.

During non-reducing native PAGE analysis, E2 was predominantly detected as a dimer, whereas an E1–E2 heterodimer band was not observed. This observation may be related to the structural properties of pestiviral envelope glycoproteins. Previous studies have shown that E1–E2 interactions can depend on membrane association and the native endoplasmic reticulum environment [38,40]. In the present system, truncation of the E2 transmembrane region and expression in a recombinant context may affect the stability or detectability of the E1–E2 heterodimer. Therefore, the absence of a detectable heterodimer band in native PAGE does not necessarily indicate the absence of E1–E2 interactions. Instead, the restoration of E2 expression and antigenicity upon E1 co-expression, together with immunofluorescence and immunogenicity data, is consistent with a possible role for E1 in facilitating proper folding and stabilization of E2.

From a vector performance perspective, insertion of the E1E2 expression cassette did not compromise key biological properties of MVA. Recombinant viruses exhibited growth kinetics indistinguishable from parental MVA in permissive DF-1 cells and BHK-21 cells, retained typical virion morphology, and maintained genetic stability after serial passaging in vitro. These features are consistent with the established safety and manufacturing advantages of MVA and support the feasibility of rMVA-BVDV1-E1E2 and rMVA-BVDV2-E1E2 for scalable vaccine production [44,45,46,47]. MVA has several advantages as a vaccine vector, including its safety profile, large insert capacity, and ability to induce both humoral and cellular immune responses without reversion to virulence. Unlike adenoviral or lentiviral vectors, MVA has a low risk of pre-existing immunity in the target population and is well-suited for large-scale vaccine production, making it a potentially useful platform for veterinary vaccine development [20,47,48,49].

Immunogenicity analyses demonstrated that rMVA-BVDV1-E1E2 and rMVA-BVDV2-E1E2 induced detectable humoral immune responses in mice, characterized by the presence of E2-specific IgG antibodies and virus-neutralizing activity following booster immunization. The neutralizing antibody titers observed in rMVA-E1E2 immunized mice were broadly comparable to those induced by the inactivated vaccine in this mouse model, indicating that the recombinant construct is capable of eliciting measurable antibody responses. Although heterologous neutralization titers were lower than those against the homologous virus, the presence of detectable cross-neutralizing activity suggests partial antigenic overlap between BVDV-1 and BVDV-2 E2 proteins. Analysis of IgG subclasses showed higher IgG2a levels relative to IgG1 in most groups, resulting in IgG2a/IgG1 ratios greater than 1. This pattern suggests a tendency toward a Th1-biased immune response, a profile frequently reported for poxvirus vectored vaccines [50,51,52,53,54]. However, the magnitude of the subclass differences was moderate and should be interpreted cautiously, particularly in the absence of additional functional assays.

In addition to humoral immunity, rMVA-BVDV1-E1E2 and rMVA-BVDV2-E1E2 induced detectable antigen-specific cellular immune responses. Following in vitro stimulation with E2 antigen, splenocytes from immunized mice showed an increased proportion of CD8^+^ T cells compared with the MVA control group as determined by flow cytometry, together with higher frequencies of IFN γ–secreting cells detected by ELISpot assay. Although these findings indicate activation of cellular immune pathways, a defined protective threshold for ELISpot responses in BVDV infection has not been established, and therefore the relationship between these responses and protective immunity remains to be determined. Nevertheless, the induction of antigen-responsive T cells is consistent with the known immunostimulatory properties of MVA vectors [55,56].

Several limitations of the present study should be acknowledged. Protective efficacy against virulent BVDV challenge was not evaluated, and immunogenicity was assessed exclusively in a murine model rather than in the natural bovine host. The mouse model used in this study therefore provides only a preliminary evaluation of immunogenicity. Future studies in cattle, including challenge experiments, will be necessary to determine the protective efficacy of rMVA-E1E2 and its ability to prevent clinical disease and viral transmission in the natural host. In addition, comparative evaluations against licensed BVDV vaccines would provide valuable insight into the relative advantages of this platform.

In summary, we report the successful development of a genetically stable recombinant MVA co-expressing BVDV E1 and E2 that retains favorable biological properties and induces neutralizing antibody responses together with antigen-specific CD8^+^ T-cell activation in mice. By addressing the structural dependence of E2 on E1, this study provides additional insight into BVDV antigen design and suggests the potential feasibility of E1–E2 co-expression as a vaccine strategy.

## 5. Conclusions

This study investigated the feasibility of co-expression of BVDV E1 and E2 using a Modified Vaccinia Virus Ankara vector as an alternative strategy to address some limitations of current BVDV vaccines. By considering the structural relationship between E1 and E2, rMVA-BVDV1-E1E2 and rMVA-BVDV2-E1E2 allowed detectable antigen expression in the recombinant system and induced measurable humoral and cellular immune responses in a mouse proof-of-concept model, including virus-neutralizing antibodies and antigen-responsive CD8^+^ T cell activation, while retaining key biological properties of MVA such as genetic stability and a favorable safety profile. These findings provide preliminary experimental support for the use of E1–E2 co-expression in MVA-based vaccine design. Although further evaluation in the natural bovine host, including challenge studies, will be required, the present results support further investigation of MVA-based E1E2 constructs as a potential platform for BVDV vaccine development and highlight the importance of antigen structural context in viral vaccine design.

## Data Availability

The data presented in this study are available within the article and Supplementary Material.

## Supplementary Materials

The following supporting information can be downloaded at: https://www.mdpi.com/article/10.3390/vaccines14040337/s1, Figure S1: Expression analysis of BVDV E2 protein expressed alone in recombinant MVA; Figure S2: Original uncropped blots corresponding to Figure 2; Figure S3: Original immunofluorescence images corresponding to Figure 2C; Figure S4: Original immunofluorescence images corresponding to Figure 2D.
