# Supplementary material for: Construction and Immunogenicity of Modified Vaccinia Ankara (MVA) Viruses Expressing E1 and E2 Proteins of Bovine Viral Diarrhea Virus
Source: Vaccines (Basel). 2026 Apr 11;14(4):337. doi: 10.3390/vaccines14040337 (PMC13120248; doi:10.3390/vaccines14040337)
Supplement: Supplementary file 1 [file vaccines-14-00337-s001.zip › vaccines-4230560-supplementary.pdf]

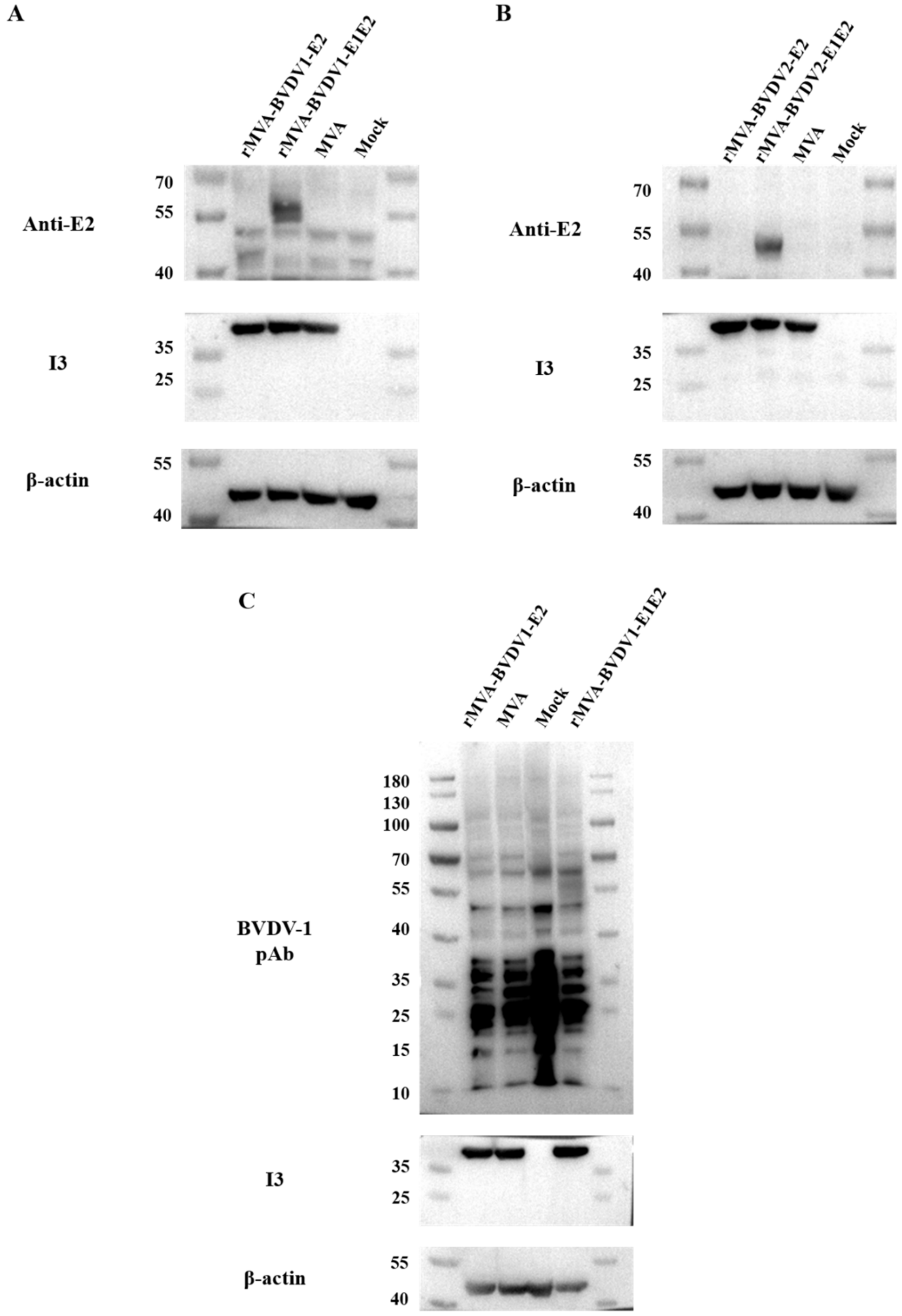

Figure S1. Expression analysis of BVDV E2 protein expressed alone in recombinant MVA. **(A)** Western blot analysis of BVDV-1 E2 expressed alone in recombinant MVA. BHK-21 cells were mock-infected or infected with purified parental MVA or recombinant MVA expressing BVDV-1 E2 alone at 3 PFU/cell for 24 h. Cell lysates were subjected to SDS-PAGE and immunoblotted with the same BVDV E2-specific monoclonal antibodies used in Figure 2A. β-actin was used as a loading control. No specific E2 band was detected under these conditions. **(B)** Western blot analysis of BVDV-2 E2 expressed alone in recombinant MVA. BHK-21 cells were mock-infected or infected with purified parental MVA or recombinant MVA expressing BVDV-2 E2 alone at 3 PFU/cell for

24 h. Cell lysates were subjected to SDS-PAGE and immunoblotted with the same BVDV E2-specific monoclonal antibodies used in Figure 2B. β-actin was used as a loading control. No detectable E2 protein signal was observed. **(C)** Detection of BVDV-1 E2 using BVDV-1 polyclonal antibodies. BHK-21 cells were mock-infected or infected with recombinant MVA expressing BVDV-1 E2 alone at 3 PFU/cell for 24 h. Cell lysates were analyzed by Western blot using BVDV-1 polyclonal antibodies. β-actin was used as a loading control. No specific E2 signal was detected. These results suggest that E2 expressed alone was not detectable under the experimental conditions used in this study. Anti-I3 MmAb(gifts from Bernard Moss).

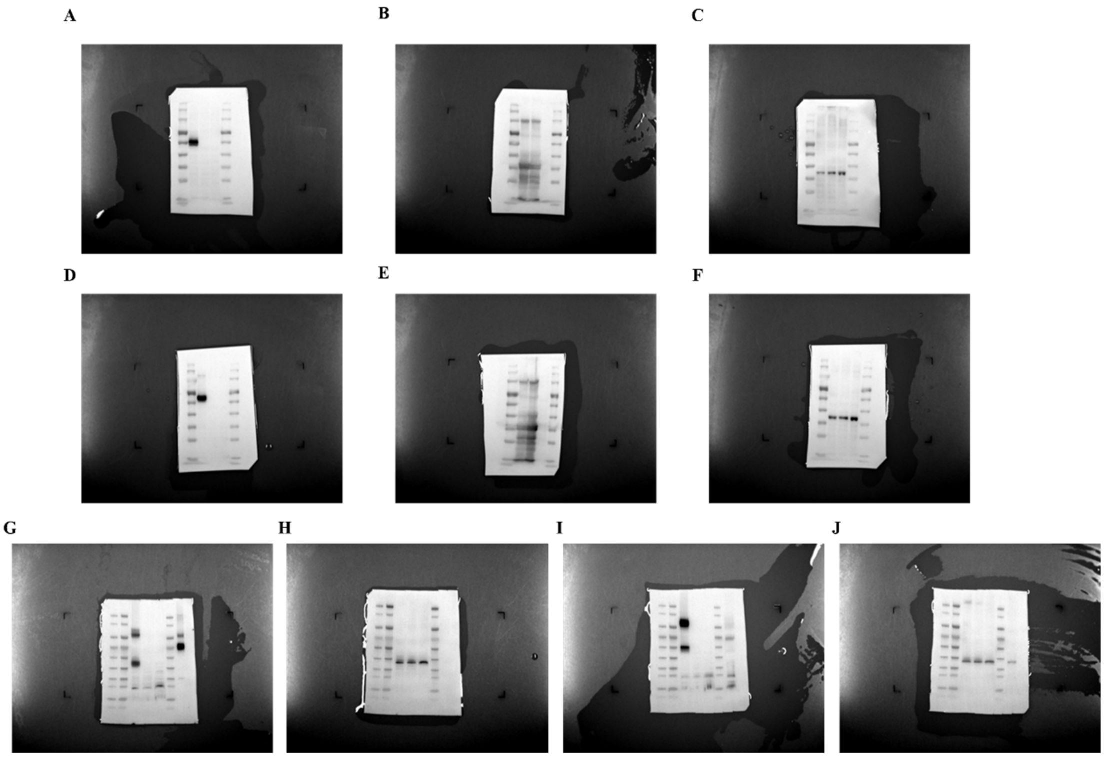

Figure S2. Original uncropped blots corresponding to Figure 2. Expression of the E2 protein in recombinant MVA. **(A–F)** Raw western blot images for Figure 2A. BHK-21 cells were mock-infected or infected with purified parental MVA or recombinant rMVA-BVDV1-E1E2 or rMVA-BVDV2-E1E2 at 3 PFU/cell for 24 h. Cell lysates were subjected to SDS-PAGE and immunoblotted with antibodies against BVDV E2, vaccinia virus (VACV), and GAPDH, as indicated. **(G–J)** Raw western blot images for Figure 2B. BHK-21 cells were mock-infected or infected with purified parental MVA or recombinant rMVA-BVDV1-E1E2 or rMVA-BVDV2-E1E2 at 3 PFU/cell for 24 h. Cell lysates were analyzed under non-reducing conditions by native-PAGE followed by western blotting using antibodies against BVDV E2 and GAPDH, as indicated. E2 monomer and E2 dimer correspond to those shown in Figure 2B.

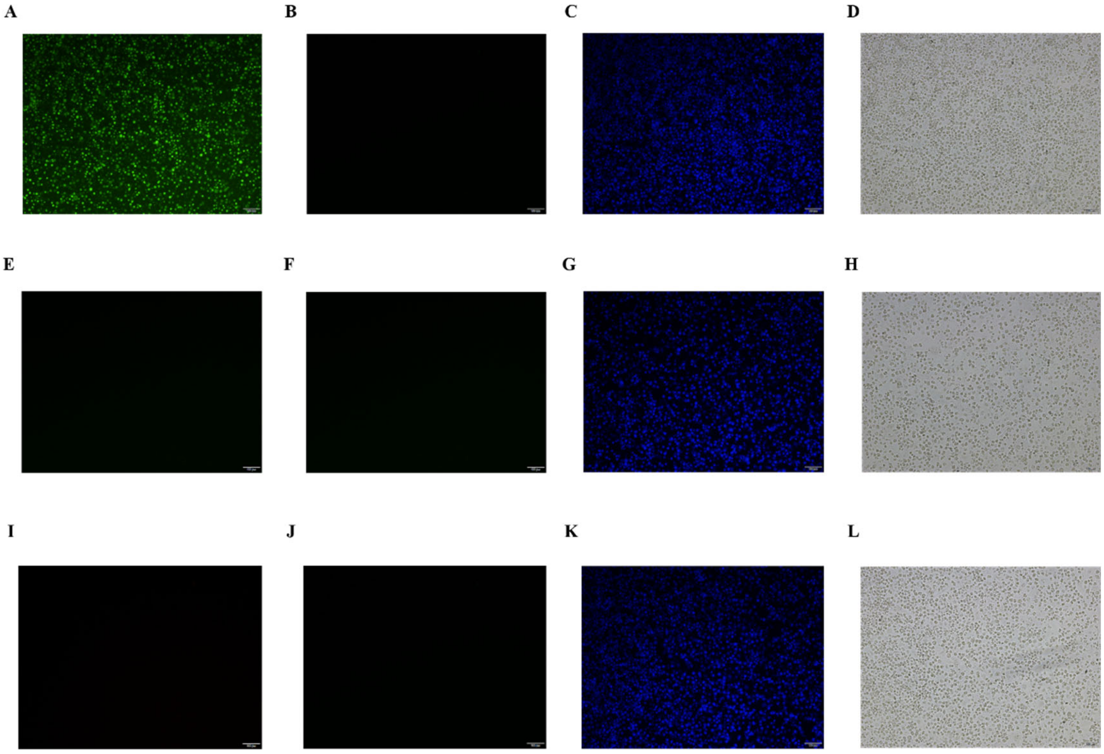

Figure S3. Original immunofluorescence images corresponding to Figure 2C. Immunofluorescence analysis of E2 protein expression in rMVA-BVDV1-E1E2–infected cells. **(A–D)** BHK-21 cells infected with rMVA-BVDV1-E1E2 showing FITC (E2, green), mCherry, DAPI (nuclei, blue), and bright-field images. **(E–H)** BHK-21 cells infected with parental MVA showing FITC, mCherry, DAPI, and bright-field images. **(I–L)** Mock-infected BHK-21 cells showing FITC, mCherry, DAPI, and bright-field images. Cells were treated under the same conditions as described in Figure 2C. At 24 h post-infection, cells were fixed, permeabilized, and blocked. Intracellular E2 was detected using specific primary antibodies followed by FITC-conjugated secondary antibodies (green), and nuclei were counterstained with Hoechst (blue).

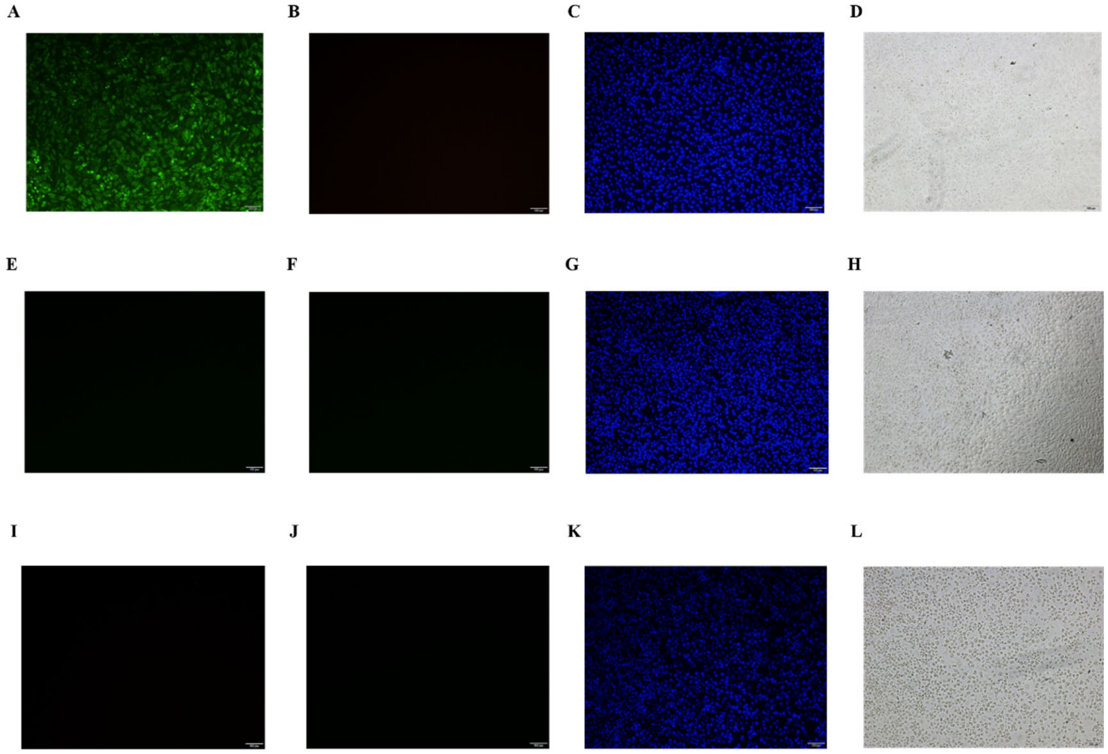

Figure S4. Original immunofluorescence images corresponding to Figure 2D. Immunofluorescence analysis of E2 protein expression in rMVA-BVDV2-E1E2–infected cells. **(A–D)** BHK-21 cells infected with rMVA-BVDV2-E1E2 showing FITC (E2, green), mCherry, DAPI (nuclei, blue), and bright-field images. **(E–H)** BHK-21 cells infected with parental MVA showing FITC, mCherry, DAPI, and bright-field images. **(I–L)** Mock-infected BHK-21 cells showing FITC, mCherry, DAPI, and bright-field images. Cells were treated under the same conditions as described in Figure 2D. At 24 h post-infection, cells were fixed, permeabilized, and blocked. Intracellular E2 was detected using specific primary antibodies followed by FITC-conjugated secondary antibodies (green), and nuclei were counterstained with Hoechst (blue).
